# Supplementary material for: A novel method for assessing and measuring homophily in networks through second-order statistics
Source: Sci Rep. 2022 Jun 13;12:9757. doi: 10.1038/s41598-022-12710-7 (PMC9192693; doi:10.1038/s41598-022-12710-7)
Supplement: Supplementary file 1 — Supplementary Information 1. [file 41598_2022_12710_MOESM1_ESM.pdf]

Appendix

Basic statistic tools

In this section, after some preliminaries, we show how to compute probabilities of events related to random **c**-colorings and used throughout the paper.

For a positive integer number a and a nonnegative integer number r, the *r-th falling factorial of a* (also referred to as *r-th falling power of a* in Knuth's terminology [14]) is the number:

$$a^{\underline{r}} = \frac{a!}{(a-r)!} = \underbrace{a(a-1)\cdots(a-r+1)}_{r \text{ factors}}$$

and it counts the number of injective mapping from a set of r elements into a set of a elements. One has

- $a^{\underline{r}} = 0$ if $r > a$;
- $a^{\underline{0}} = 1$, $a^{\underline{1}} = a$ and $a^{\underline{a}} = a!$;
- $a^{\underline{r+s}} = a^{\underline{r}}(a-r)^{\underline{s}}$

The reason for Knuth's "falling power" terminology in now clear. Let us come back to the definition of **c**-coloring which we recall here: let V be a set with n elements and, for a positive integer s, let $\mathbf{c} = (c_1, \ldots, c_s)$ be a weak composition of n, namely an order sensitive non negative integer vector whose entries add up to n. A **c**-coloring of V is a surjective map $f : V \to [s]$ such that, for each $i \in [s]$ each *color class* $f^{-1}(i)$ has exactly c_i elements; **c** is the *profile of f*. The *multinomial coefficient* with *parts* $c_1, c_2 \cdots c_s$

$$\binom{n}{\mathbf{c}} = \binom{n}{c_1 c_2 \cdots c_s} = \frac{n!}{c_1! c_2! \cdots c_s!}$$

counts the **c**-colorings of V. Indeed, the c_1 elements that are mapped to 1 can be chosen in $\binom{n}{c_1}$, the elements that are mapped to 2 can be chosen in $\binom{n-c_1}{c_2}$ among the remaining $n - c_1$. Continuing in this way and taking the product of these binomial coefficients we obtain the expression above. Note that, for $s = 2$, the multinomial coefficient with parts c_1 and c_2 (with $c_2 = n - c_1$), reduces to the binomial coefficient:

$$\binom{n}{c_1 c_2} = \binom{n}{c_1} = \binom{n}{c_2}.$$

Also recall that the binomial coefficient $\binom{n}{r}$ is defined for any pair of positive integers n and r as follows,

$$\binom{n}{r} = \begin{cases} \frac{n!}{r!(n-r)!} & \text{if } 0 \le r \le n \\ 0 & \text{otherwise.} \end{cases}$$

Let $J \subseteq [s]$. The *contraction by J* of vector $\mathbf{c} = (c_1, \ldots, c_t)$ is the vector $\mathbf{c}'$ obtained from $\mathbf{c}$ by suppressing the entries whose indices are in J. We make use of the following multinomial identity which follows straightforwardly by the definition of the multinomial coefficient:

$$\binom{n}{\mathbf{c}} = \left(\prod_{j \in J} \binom{n}{c_j} \right) \cdot \binom{n - \sum_{j \in J} c_j}{\mathbf{c}'}, \qquad (1)$$

where n and $\mathbf{c}$ are as above and $\mathbf{c}'$ is the contraction of $\mathbf{c}$ by J. For instance, if $J = \{1\}$, then $\mathbf{c}' = (c_2, \ldots, c_s)$ and the expression above reads as

$$\binom{n}{\mathbf{c}} = \binom{n}{c_1} \cdot \binom{n - c_1}{c_2 \cdots c_s}.$$

We now define the notion of random $\mathbf{c}$-colorings in some more depth. Let $\Phi(\mathbf{c}; V)$ be the set of all $\mathbf{c}$-colorings of V (in our case $V = V(G)$ for some graph G). When V is understood (as we have assumed throughout the paper) the notation is abridged into $\Phi(\mathbf{c})$. Thus

$$\Phi(\mathbf{c}) = \{f : V \to [s] \mid f \text{ is surjective}\}.$$

We now equip $\Phi(\mathbf{c})$ with the uniform measure $\mathbb{P}_{n,\mathbf{c}}$

$$\mathbb{P}_{n,\mathbf{c}}(f) = |\Phi(\mathbf{c})|^{-1} = \binom{n}{\mathbf{c}}^{-1}$$

and define the *random* $\mathbf{c}$*-coloring of* V, which we denote by F, as the dentity map on $\Phi(\mathbf{c})$, namely the random $\mathbf{c}$-coloring of V is essentially the probability space $(\Phi(\mathbf{c}), \mathbb{P}_{n,\mathbf{c}})$ itself and it can be visualized as the random object F taking the value $f \in \Phi(\mathbf{c})$ with probability $\Pr\{F = f\} = \mathbb{P}_{n,\mathbf{c}}(f)$. A *statistic based on the random* $\mathbf{c}$*-coloring* F *of* V is simply any measurable function on $(\Phi(\mathbf{c}), \mathbb{P}_{n,\mathbf{c}})$, for instance, the indicator X_v^i of the event $(F(v) = i)$, for some $i \in [s]$ and $v \in V$, is one of such. Notice that the inverse image of event $(F(v) = i)$ is the set $\{f \in \Phi(\mathbf{c}) \mid f(v) = i\}$. This is the essence of our statistical model.

For our purposes, for some two disjoint subset A and B of V and some color $i \in [s]$, we are interested in the probability of the event that all the elements of A have color i while all those of B have not. Let $\Omega_i(A, B)$ denote this event. Hence

$$\begin{aligned}\Pr\{\Omega_i(A, B)\} &= \Pr\{(F(a) = i,\ \forall a \in A) \wedge (F(b) \neq i,\ \forall b \in B)\}\\ &= \left|\{f \in \Phi_{\mathbf{c}} \mid A \subseteq f^{-1}(i) \subseteq V \setminus B\}\right| \Big/ \binom{n}{\mathbf{c}}.\end{aligned}$$

We are also interested in computing the probability of the intersection of two such events for two distinct colors. We summarize these calculations in the next lemma and then we show how to use the lemma for computing the probability of certain simpler events.

Lemma 1 *Let A, A', B, B' be subsets of V and let a, a', b, b' be their respective cardinalities. Suppose $A \cap B = \emptyset$, $A' \cap B' = \emptyset$, $A \cap A' = \emptyset$ and $B \cap B' = \emptyset$ and let $b'' = |B' \cap A|$. Then, for each two distinct colors i and j, one has*

$$\Pr\{\Omega_i(A, B) \wedge \Omega_j(A', B')\} = \left\{\frac{c_i^{\underline{a}}(n - c_i)^{\underline{b}}}{n^{\underline{a+b}}}\right\}\left\{\frac{c_j^{\underline{a'}}(n - c_i - c_j)^{\underline{b'-b''}}}{(n - c_i)^{\underline{a'+(b'-b'')}}}\right\} \tag{2}$$

Proof. Since the elements of A have to be mapped to i and those of B have not, the elements that have color i can be chosen in $\binom{n-(a+b)}{c_i-a}$ ways. After this choice, we are left with $n - c_i$ elements that have to be assigned to $[s] \setminus \{i\}$ in such a way that all the elements in A' must be mapped to j and those in B' cannot. Among the elements of B' the are possibly some that have been already assigned to i. Therefore we can perform the choice in $\binom{n-c_i-(a'+b'-b'')}{c_j-a'}$ ways. After this choice has been done, we are left with $n - (c_i + c_j))$ elements that have to be assigned to colors in $[s] \setminus \{i, j\}$, namely with the number of $\mathbf{c}'$-colorings of a set of $n - (c_i + c_j))$ elements where $\mathbf{c}'$ is the contraction of $\mathbf{c}$ by $\{i, j\}$. If follows that

$$\Pr\{\Omega_i(A, B) \wedge \Omega_j(A', B')\} = \frac{\binom{n-(a+b)}{c_i-a}\binom{n-c_i-(a'+b'-b'')}{c_j-a'}\binom{n-c_i-c_j}{\mathbf{c}'}}{\binom{n}{c_i}\binom{n-c_i}{c_j}\binom{n-c_i-c_j}{\mathbf{c}'}},$$

where we used Formula (1) at the denominator. One obtains Formula (2) after simplifying, expanding the binomial coefficients and resorting to the definition of falling factorial. □

The way we use the lemma to compute the probability of certain basic events is to read the events as a special case of the event $\Omega_i(A, B) \wedge \Omega_j(A', B')$ and to plug in the formula the corresponding parameters $a, b, \ldots b''$. Note that, for any $j \in [s]$, by choosing $A' = B' = \emptyset$ (amd $a' = b' = b'' = 0$ correspondingly) makes the event $\Omega_j(A', B')$ almost sure. Hence

$$\begin{aligned}\Pr\{(F(a) = i,\ \forall a \in A) \wedge (F(b) \neq i,\ \forall b \in B)\} &= \Pr\{\Omega_i(A, B)\}\\ &= \Pr\{\Omega_i(A, B) \wedge \Omega_j(\emptyset, \emptyset)\}\\ &= \frac{c_i^{\underline{a}}(n - c_i)^{\underline{b}}}{n^{\underline{a+b}}} = \frac{c_i^{\underline{a}}}{n^{\underline{a}}}\frac{(n - c_i)^{\underline{b}}}{(n - a)^{\underline{b}}}.\end{aligned} \tag{3}$$

By, taking $B = \emptyset$—and hence $b = 0$–has the effect of suppressing the constraint $(F(b) \neq i, \forall b \in B)$. Therefore, for instance,

$$\Pr\{F(a) = i, \forall a \in A\} = \Pr\{\Omega_i(A, \emptyset)\} = \Pr\{\Omega_i(A, \emptyset) \wedge \Omega_j(\emptyset, \emptyset)\} = \frac{c_i^{\underline{a}}}{n^{\underline{a}}} \tag{4}$$

and, in particular, for any pair of elements $u, v \in V$ any color $i \in [s]$,

$$\Pr\{F(u) = i\} = \frac{c_i^{\underline{1}}}{n^{\underline{1}}} = \frac{c_i}{n} \quad \text{and} \quad \Pr\{(F(u) = i) \wedge (F(v) = i)\} = \frac{c_i^{\underline{2}}}{n^{\underline{2}}} = \frac{c_i(c_i - 1)}{n(n-1)}. \tag{5}$$

Analogously, since for any pair of elements $u, v \in V$ and any two distinct colors $i, j \in [s]$, it holds that

$$(F(u) = i) \wedge (F(v) = j) = \Omega_i(\{u\}, \{v\}) \wedge \Omega_j(\{v\}, \{u\})$$

it follows that two compute the probability of such an event one has to put $a = b = a' = b' = b'' = 1$ in Formula (2) to obtain

$$\Pr\{(F(u) = i) \wedge (F(v) = j)\} = \frac{c_i^{\underline{1}} c_j^{\underline{1}}}{n^{\underline{2}}} = \frac{c_i c_j}{n(n-1)}. \tag{6}$$

Proof of Theorem 1

Proof. The expected values $\overline{m}_{i,i}$ and $\overline{m}_{i,j}$, $i \neq j$ have already been computed. Let us prove the formula for the expected value of L^i. By definition W_v^i is the indicator of the event $(F(v) = i) \wedge (F(w) \neq i, \forall w \in N_G(v))$, namely the event that v has color i while all of its neighbors have not. Thus, after (4),

$$\mathbb{E}\left(W_v^i\right) = \Pr\left\{W_v^i = 1\right\} = \Pr\left\{X_v^i = 1, D_{N_G(v)}^i = 0\right\} = \frac{c_i}{n} \Pr\left\{D_{N_G(v)}^i = 0 \middle| X_v^i = 1\right\} = \frac{c_i}{n} \cdot \frac{(n - c_i)^{\underline{\deg_G(v)}}}{(n-1)^{\underline{\deg_G(v)}}}.$$

Hence, by linearity of expectation

$$\mathbb{E}\left(L^i\right) = \frac{c_i}{n} \sum_{v \in V(G)} \frac{(n - c_i)^{\underline{\deg_G(v)}}}{(n-1)^{\underline{\deg_G(v)}}}.$$

Let us compute the variance of the random variables in 1), 2) and 3). Observe that all such variables are sums of Bernoulli random variables, namely they are of the form $S = \sum_{\nu \in N} B_\nu$ where N is a finite index set and B_ν is a Bernoulli random variable for each index $\nu \in N$. The variance of S is thus given by

$$\begin{aligned}
\operatorname{var}(S) = \mathbb{E}\left(S^2\right) - (\mathbb{E}(S))^2 &= \mathbb{E}\left(\left(\sum_{\nu \in N} B_\nu\right)^2\right) - (\mathbb{E}(S))^2 = \\
&= \mathbb{E}\left(\sum_{\nu \in N} B_\nu\right) + \mathbb{E}\left(\sum_{\substack{(\nu, \nu') \in N \times N \\ \nu \neq \nu'}} B_\nu B_{\nu'}\right) - (\mathbb{E}(S))^2 = \\
&= \mathbb{E}(S)(1 - \mathbb{E}(S)) + \sum_{\substack{(\nu, \nu') \in N \times N \\ \nu \neq \nu'}} \mathbb{E}(B_\nu B_{\nu'}) = \\
&= \mathbb{E}(S)(1 - \mathbb{E}(S)) + \sum_{\substack{(\nu, \nu') \in N \times N \\ \nu \neq \nu'}} \Pr\{B_\nu = 1 \wedge B_{\nu'} = 1\}
\end{aligned} \tag{7}$$

where we used the fact that $B_\nu = B_\nu^2$ and that $\mathbb{E}(B_\nu B_{\nu'}) = \Pr\{B_\nu = 1 \wedge B_{\nu'} = 1\}$. Let us first specialize the formula above to $M^{i,i}$ and $M^{i,j}$. Notice that in both cases $N = E(G)$ and that the summation set in the last equality of (7) is $E(G) \times E(G) \setminus \{(e, e) \mid e \in E(G)\}$. Denote the latter set by P. Since two edges e and e' of G can have at most one node in common, it follows that $P = Q \cup R$ where

$Q = \{(e, e') \in P \mid e \sim e'\}$ and $R = \{(e, e') \in P \mid e \not\sim e'\}$ and where we have written $e \sim e'$ if e and e' share a node and $e \not\sim e'$ otherwise. Clearly $Q \cap R = \emptyset$. Therefore, if S is either $M^{i,i}$ or $M^{i,j}$, the variance of S is

$$\mathrm{var}(S) = \mathbb{E}(S)(1 - \mathbb{E}(S)) + \sum_{Q} \Pr\{B_e = 1 \wedge B_{e'} = 1\} + \sum_{R} \Pr\{B_e = 1 \wedge B_{e'} = 1\}.$$

It is clear that $\Pr\{B_e = 1 \wedge B_{e'} = 1\}$ assumes only two values over the set P: it assumes the value a on Q, and the value b on R. Moreover, since $|P| = (m^2 - m) = 2\binom{m}{2}$ and since $e \sim e'$ if and only if e and e' spans a P_3, it follows that

$$|Q| = 2\pi_3(G) = 2 \sum_{v \in V(G)} \binom{\deg_G(v)}{2} \quad \text{and} \quad |R| = 2\binom{m}{2} - 2\pi_3(G).$$

Therefore, the variance of S assumes the following form

$$\mathrm{var}(S) = \mathbb{E}(S)(1 - \mathbb{E}(S)) + 2\left[\pi_3(G)(a - b) + \binom{m}{2} b\right]. \tag{8}$$

We obtain expressions for the variance of $M^{i,i}$ and $M^{i,j}$ by plugging the expectation of the corresponding variable in the formula above and specializing a and b for $B_e = Y_e^{i,j}$ and $B_e = Y_e^{i,j}$, with $e = uv$ for some nodes u and v.

Let us start with a, namely, the value of $\Pr\{B_e = 1 \wedge B_{e'} = 1\}$ when $(e, e') \in Q$. Hence $e \sim e'$. After regarding edges as sets of two nodes, one has $e \sim e'$ if and only if $|e \cup e'| = 3$ (recall that the graph is loopless and has no parallel edges). Let $e \cup e' = \{u, v, w\}$ where u is the unique node in $e \cap e'$. Recall that for disjoint subsets A and B of $V(G)$ we denote by $\Omega_i(A, B)$ the event that all the nodes of A have color i while all those of B have not. Now, if $S = M^{i,i}$, then $B_e = Y_e^{i,i}$ for all $e \in E(G)$, and thus $a = \Pr\{\Omega_i(\{u, v, w\}, \emptyset)\}$; else, if $S = M^{i,j}$, then $B_e = Y_e^{i,j}$ for all $e \in E(G)$; in this case observe a is the sum of the probability of two mutually exclusive events: the event that u has color i while the nodes in $\{v, w\}$ have color j, namely the event $\Omega_i(\{u\}, \{v, w\}) \wedge \Omega_j(\{v, w\}, \{u\})$, and the the event that u has color j while the nodes in $\{v, w\}$ have color i, namely the event $\Omega_j(\{u\}, \{v, w\}) \wedge \Omega_i(\{v, w\}, \{u\})$. Therefore, by Lemma 1, one has

$$a = \begin{cases} \frac{c_i^3}{n^3} & \text{if } B_e = Y_e^{i,j} \\ \frac{c_i c_j^2 + c_i^2 c_j}{n^3} & \text{if } B_e = Y_e^{i,j} \end{cases}.$$

Let us compute b. In this case $e \cap e' = \emptyset$. Let $e = uv$ and $e' = u'v'$. If $S = M^{i,i}$, then $B_e = Y_e^{i,i}$ for all $e \in E(G)$, and thus a is the probability of the event $\Omega_i(\{u, u', v, v'\}, \emptyset)$, namely the probability that all the four nodes have color i under F; else, if $S = M^{i,j}$, then $B_e = Y_e^{i,j}$ for all $e \in E(G)$; observe that there are two bipartitions of $\{u, u', v, v'\}$ into sets A and B such that $|A| = |B| = 2$ and neither A nor B induces one of the edges e and e'. Hence b is two times the probability that all the nodes in A have one of the colors i or j and all the nodes in B have the other color. Hence b is four times the probability of the event that all nodes in A have color i and all nodes in B have color j, that is $b = 4\Pr\{\Omega_i(A, B) \wedge \Omega_j(B, A)\}$. Therefore, still by Lemma 1, one has

$$b = \begin{cases} \frac{c_i^4}{n^4} & \text{if } B_e = Y_e^{i,i} \\ 4\frac{c_i^2 c_j^2}{n^4} & \text{if } B_e = Y_e^{i,j} \end{cases}.$$

By plugging the values of a and b (as well as the corresponding expected values) in (8) one achieves the desidered expressions for $\sigma_{i,i}^2$ and $\sigma_{i,j}^2$. It only remains to prove the formula for the variance of L^i. By specializing (7) with $S = L^i$, $N = V(G)$, $B_v = W_v^i$ one gets

$$\mathrm{var}(L^i) = \mathbb{E}(L^i)(1 - \mathbb{E}(L^i)) + \sum_{\substack{(u,v) \in V(G) \\ u \neq v}} \Pr\{W_u^i = 1, W_v^i = 1\}$$

and since $\Pr\left\{W_u^i = 1, W_v^i = 1\right\} = 0$ whenever u and v are adjacent nodes of G, it follows that

$$\mathrm{var}(L^i) = \mathbb{E}\left(L^i\right)\left(1 - \mathbb{E}\left(L^i\right)\right) + \sum_{\substack{(u,v)\in V(G)\\ u\neq v, uv\notin E(G)}} \Pr\left\{\left(X_u^i = 1, X_v^i = 1\right) \wedge \left(D^i_{N_G(u)\cup N_G(v)} = 0\right)\right\}.$$

Hence, after setting $b(u,v) = |N_G(u)\cup N_G(v)| = \deg_G(u) + \deg_G(v) - |N_G(u)\cap N_G(v)|$, by (3) with $a = 2$ and $b = b(u,v)$ it follows that

$$\Pr\left\{\left(X_u^i = 1, X_v^i = 1\right) \wedge \left(D^i_{N_G(u)\cup N_G(v)} = 0\right)\right\} = \frac{c_i^{\underline{2}}}{n^{\underline{2}}} \frac{(n - c_i)^{\underline{b(u,v)}}}{(n-2)^{\underline{b(u,v)}}}$$

and after plugging this expression in the latter sum we obtain the stated formula. The proof is thus completed. □

Classes' size in organism's networks

For each organism's network, we report in Table 1 the number of nodes for each functional class, and the total number of nodes. Nodes in classes A, B, Y, and Z are included in the total size, but were not considered in the analisys.

Species	Bm	Ec	Hi	Hp	Mt	Sp	Tp	Vc	Pa	Sc
C	158	203	89	64	168	43	34	151	113	133
D	26	27	23	18	38	20	12	32	16	53
E	298	262	136	86	185	132	20	216	120	172
F	60	64	51	33	63	60	21	65	47	80
G	143	215	98	29	104	174	41	135	69	147
H	114	109	65	65	119	43	19	121	58	95
I	90	65	41	38	129	32	17	67	18	81
J	153	131	140	118	141	136	113	159	146	336
K	107	135	69	22	123	104	26	133	74	143
L	110	118	100	81	155	101	58	133	51	131
M	138	156	110	82	99	81	59	144	43	42
N	33	72	6	42	9	5	43	90	27	5
O	110	99	76	62	92	48	42	104	43	212
P	105	137	80	42	103	64	22	133	62	76
Q	34	23	13	8	78	7	1	35	10	23
T	60	56	32	15	70	39	20	77	13	79
U	29	33	23	35	18	17	11	35	10	78
V	35	38	16	24	36	54	7	40	21	9
X	871	2076	440	400	2048	651	328	1281	619	4169
**total nodes**	**2675**	**4020**	**1609**	**1264**	**3779**	**1811**	**894**	**3153**	**1564**	**6157**

Table 1: For each organisms, the total number of nodes (classes A, B, Y, Z included) and the number of nodes in each considered functional class.

Speeding-up L^i computation

We show how to compute efficiently statistics in point 3) in Theorem 1, in particular the variance expression

$$\mathrm{var}(L^i) = \mathbb{E}\left(L^i\right)\left(1 - \mathbb{E}\left(L^i\right)\right) + \frac{c_i^{\underline{2}}}{n^{\underline{2}}} \sum_{\substack{(u,v)\in V(G)\\ u\neq v, uv\notin E(G)}} \frac{(n - c_i)^{\underline{b(u,v)}}}{(n-2)^{\underline{b(u,v)}}} \tag{9}$$

Trivially computing the summation in (9) requires $O(n^3)$ time. We show now that the time complexity can be lowered to $O\left(\sum_{u\in V(G)} \deg_G^3(u)\right)$, that becomes $O\left(\sum_{u\in V(G)} \deg_G^2(u)\right)$ expected time (with very high probability) if hash-tables are used to represent sets, and falling factorial values $x^{\underline{y}}$ are approximated by applying Stirling formula. Since huge networks are usually very sparse, this represents a deep improvement with respect to the computation based on (9).

We first observe that

$$\sum_{\substack{(u,v)\in V(G)\\ u\neq v, uv\notin E(G)}} \frac{(n-c_i)^{\underline{b(u,v)}}}{(n-2)^{\underline{b(u,v)}}} =$$

$$= \sum_{(u,v)\in V(G)} \frac{(n-c_i)^{\underline{b(u,v)}}}{(n-2)^{\underline{b(u,v)}}} - 2\sum_{uv\in E(G)} \frac{(n-c_i)^{\underline{b(u,v)}}}{(n-2)^{\underline{b(u,v)}}} - \sum_{u\in V(G)} \frac{(n-c_i)^{\underline{b(u,u)}}}{(n-2)^{\underline{b(u,u)}}} \tag{10}$$

The second and third summations in (10) contain respectively only $O(m)$ and $O(n)$ terms. The first summation in (10) contains $O(n^2)$ terms, and for each pair u, v a different value of exponent $b(u, v)$ could be needed. This actually only occurs for pairs u, v having some common neighbor, while if all pairs had distance larger than 2 a substantial speed-up could be possible. We actually compute the first summation in (10) as if all pairs u, v had no common neighbors, so that $b(u, v) = \deg_G(u) + \deg_G(v)$, and then we fix the correct value for pairs u, v such that $\mathrm{dist}(u, v) = 2$—adjacent pairs have already been taken into account in the second summation.

Let us denote $\deg_G(u) + \deg_G(v)$ by $b'(u, v)$:

$$\sum_{(u,v)\in V(G)} \frac{(n-c_i)^{\underline{b(u,v)}}}{(n-2)^{\underline{b(u,v)}}} =$$

$$= \sum_{(u,v)\in V(G)} \frac{(n-c_i)^{\underline{b'(u,v)}}}{(n-2)^{\underline{b'(u,v)}}} + \sum_{\substack{(u,v)\in V(G)\\ \mathrm{dist}(u,v)=2}} \left(\frac{(n-c_i)^{\underline{b(u,v)}}}{(n-2)^{\underline{b(u,v)}}} - \frac{(n-c_i)^{\underline{b'(u,v)}}}{(n-2)^{\underline{b'(u,v)}}} \right) \tag{11}$$

The first summation in (11) is easily computed by means of the *degree histogram* of G, where $\deg_G^{-1}(d)$ is the number of nodes having degree d in G:

$$\sum_{(u,v)\in V(G)} \frac{(n-c_i)^{\underline{b'(u,v)}}}{(n-2)^{\underline{b'(u,v)}}} = \sum_{\substack{0\le d_1\le n\\ 0\le d_2\le n}} \deg^{-1}(d_1)\deg^{-1}(d_2) \frac{(n-c_i)^{\underline{d_1+d_2}}}{(n-2)^{\underline{d_1+d_2}}}$$

and can be computed in $O(m)$ time, since at most $2\sqrt{m}$ distinct degree values may occur in a graph. The second summation in (11) can be computed by exploring the neighborhood of each node, since $\mathrm{dist}(u, v) = 2$ if and only if $u, v \in N_G(z)$ for some node z and $uv \notin E(G)$; this can be done in $O\left(\sum_{z\in V(G)} \deg^2(z)\right)$, that is much smaller that n^2 for sparse graphs. It is immediate to see that $O\left(\sum_{z\in V(G)} \deg^2(z)\right)$ is the dominating term in computing the value of (9).

Experiments have been performed for social networks with over 10^6 nodes and $8 \cdot 10^6$ edges, for which the number of pairs of nodes u, v at distance 2 was order of 10^8.
